# Thyroid Nodule Size as a Predictor of Malignancy in Follicular and Hurthle Neoplasms

**DOI:** 10.31557/APJCP.2021.22.8.2597

**Published:** 2021-08

**Authors:** Arunnit Boonrod, Zeynettin Akkus, M Regina Castro, Atefeh Zeinodini, Kenneth Philbrick, Marius Stan, Dana Erickson, Bradley Erickson

**Affiliations:** 1 *Department of Radiology, Faculty of Medicine, Khon Kaen University, Khon Kaen, Thailand. *; 2 *Radiology Informatics Lab, Department of Radiology, Mayo Clinic, Rochester, MN, USA. *; 3 *Department of Endocrinology, Mayo Clinic, Rochester, MN USA. *

**Keywords:** Follicular neoplasm, hurthle cell neoplasm, size, talle-than-wide

## Abstract

**Introduction:**

The management of follicular (FN) and Hurthle cell neoplasms (HCN) is often difficult because of the uncertainty of malignancy risk. We aimed to assess characteristics of benign and malignant follicular and Hurthle neoplasms based on their shape and size.

**Materials and methods:**

Patients with Follicular adenoma (FA) or carcinoma (FC) and Hurthle Cell adenoma (HCA) or carcinoma (HCC) who had preoperative ultrasonography were included. Demographic data were retrieved. Size and shape of the nodules were measured. Logistic regression analyses and odds ratios were performed.

**Results:**

A total of 115 nodules with 57 carcinomas and 58 adenomas were included. Logistic regression analysis shows that the nodule height and the patient age are predictors of malignancy (p-values = 0.001 and 0.042). A cutoff value of nodule height ≥ 4 cm. produces an odds ratio of 4.5 (p-value = 0.006). An age ≥ 55 year-old demonstrates an odds ratio of 2.4-3.6 (p-value = 0.03). Taller-than-wide shape was not statistically significant (p-value = 0.613).

**Conclusion:**

FC and HCC are larger than FA and HCA in size, with a cutoff at 4 cm. Increasing age increases the odds of malignancy with a cutoff at 55 year-old. Taller-than-wide shape is not a predictor of malignancy.

## Introduction

Follicular neoplasms (FN) and Hurthle cell neoplasms (HCN) represent 2-10% of thyroid nodules and they often cause challenges in managing patients with thyroid lesions. There is not yet a true consensus criteria for triaging these patients, since the main focus of thyroid cancer is mainly on the papillary thyroid carcinoma (PTC). For both FN and HCN, cytological features that are obtained with fine-needle aspiration biopsy (FNAB) cannot accurately differentiate benign lesions from malignant ones. Because vascular or capsular invasion is required to diagnose malignancy of these types of thyroid nodules, final pathology is essential (Carling and Udelsman, 2005; Mathur et al., 2014). Surgical treatment of these nodules ranges from hemithyroidectomy to total thyroidectomy. Even with data supporting adequacy of hemithyroidectomy in 74-96% of these cases, total thyroidectomy is often performed due to cost-effectiveness, risk of reoperation if a large nodule is found to be malignant and complications of reoperation (Melck et al., 2006; Wiseman et al., 2006; Corso et al., 2014). With better preoperative predictors, proper selection of the appropriate procedure would benefit patients in terms of postoperative complications, avoidance of reoperation, and overall quality of life (Megwalu and Green, 2016; Kuba et al., 2017). Several ultrasound features, such as lack of a sonographic halo, hypoechoic appearance, predominantly solid contents, a heterogeneous echotexture, and the presence of calcifications, were reported to be predictors of follicular carcinomas (Sillery et al., 2010; Zhang and Hu, 2014). In one study volume of FC was shown to be significantly higher than FA (Sillery et al., 2010). An increasing likelihood of follicular carcinoma (FC) and Hurthle cell carcinoma (HCC) in larger nodules has been reported (Méndez et al., 2008; Gulcelik et al., 2008; Choi et al., 2009; Sillery et al., 2010; Ibrahim et al., 2015; Arpana et al., 2018; Jin et al., 2018). However, there are not many articles had compared the size of FC and HCC directly to the size of follicular adenoma (FA) and Hurthle cell adenoma (HCA) and the results are discrepancy (Seo et al., 2009; Sillery et al., 2010; Zhang and Hu, 2014). 

In this study, we evaluated the characteristics of FN and HCN based on their size, shape, and the patient data. 

## Materials and Methods

*Patients*

This study was approved by the local institutional review board with a waiver of informed consent. We retrospectively reviewed pathology reports of patients who underwent thyroidectomy at our institution between January 2012 to December 2017 to identify patients with follicular or Hurthle cell adenomas or carcinomas in the final surgical pathology. Only subjects that have preoperative US with nodules can be identified surely in both transverse and longitudinal images, were included. Demographic data and thyroid stimulating hormone (TSH) level were recorded. 

*US evaluation*

**Figure 1:**
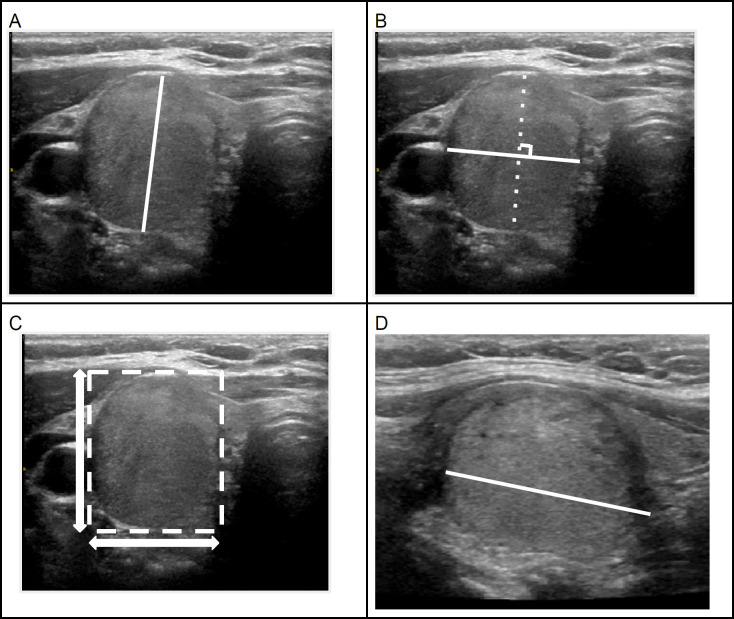
Transverse (A, B and C) and longitudinal (D) US images of an isoechoic solid thyroid nodule demonstrating the measurement method (A, B and D; depth, width and height diameters). Bounding box is drawn around the nodule (C) and the ratio between Y and X axes represents the shape of nodule. The shape of this nodule is taller-than-wide. The final pathology is a follicular adenoma

The nodules were measured in three axes by one neuroradiologist (AB). The first axis is the maximal dimension in the transverse image, the second is the maximal dimension perpendicular to the first dimension on the transverse image. These two axes are referred as width and depth, depending on the shape of the nodule. The last dimension (referred to as ‘height’) is the maximal dimension in the longitudinal image. (Tessler et al., 2017) (Figure 1) 

A taller than wide shape appearance is determined based on the transverse image comparing the diameters parallel (tallness) and perpendicular (wideness) to the ultrasound beam (Tessler et al., 2017). From this description, we drew a bounding box around the nodule and calculated the ratio between the diameters in Y-axis and X-axis of the nodule in transverse image such that the X-axis was perpendicular to the US beam and the Y-axis was parallel (Figure 1). A Y/X axis ratio greater than 1 was classified as “beam-defined” taller than wide shape. We also calculated the ratio between depth and width from the three-axes measurement. These ratios would reflect the different angulation of the nodules which might simulate the various angulations on real time US. This parameter is referred to as the “diameter-defined” ratio in our study.

*Statistical analysis*

The SPSS v.22 software package was used for statistical analysis. Univariate binary logistic regression analysis was used for both categorical data (gender, TSH categories and shape) and continuous data (age, TSH value, width, perpendicular dimension and height). For each categorical data category, the predictor value and reference category were set. The independent variables which had P-value < 0.25 were included in the next step for multivariable analysis. For multivariable analysis, forward logistic regression (LR) and backward LR methods were performed. The independent variables, which were statistically significant (P-value < 0.05) in both methods, were then analysed by enter method LR to check the multicollinearity and interactions. The area under the curve (AUC) of receiver operator characteristic (ROC) curve was used for assessing the model performance. The beam-defined and diameter ratios for taller than wide shape are compared by paired t-test and correlation determination with Pearson’s correlation.

## Results

We found 556 patients with FN and HN as the final surgical pathology in our database. Of those, 397 had preoperative US available for our review. In patients with multiple nodules, the specific location of the nodule was not stated in 162 patients, causing uncertainty of nodule localization in US and thus such cases were excluded. One hundred twenty of the patients did not have both transverse and longitudinal images. This left a total of 115 patients that could be analyzed, including 58 benign (39 FA and 19 HCA) and 57 malignant (35 FC and 22 HCC). 

The gender, age, width, depth, height and taller-than-wide appearance is found to be statistically significant (P-values = 0.031, 0.014, 0.001, 0.001, <0.001, and 0.115, respectively) based on the univariable binary logistic regression analysis of each independent variable (Table1). These variables were then analyzed by multivariable binary logistic regression analysis with forward LR and backward LR methods. In both methods, only height and age are considered statistically significant (P-values = 0.001 and 0.042, respectively) (Table 2). For visualization of P-value trend of other variables, logistic regression analysis with enter method was performed and shown in Table 2. The area under the curve (AUC) of this model is 0.72, representing a good model fit.

**Figure 2:**
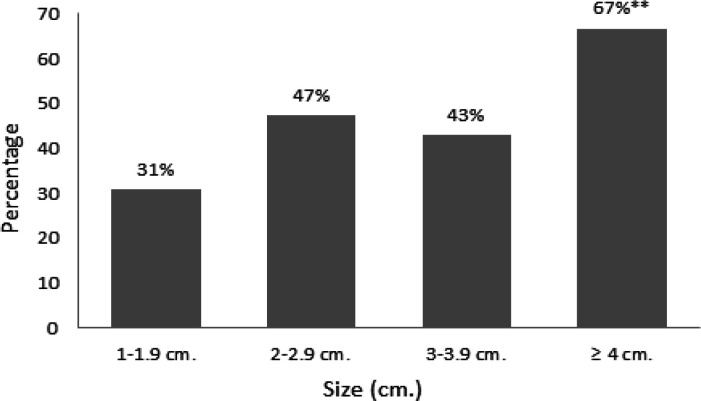
Percentage of Malignancy According to Nodule Height. **P-value = 0.006

Nodule size was stratified by height and we found that increasing size increases the odds ratio (OR) of carcinoma. The categorized size reached statistical significance at size equal to or > 4 cm (P-value = 0.006, OR = 4.5) (Table 3 and Figure 2).

Stratified age groups showed increase OR for carcinoma with increase age. The optimal cutoff value is at 55 year-old (OR = 2.4-3.6, p-value = 0.03). (Table 4).

**Figure 3:**
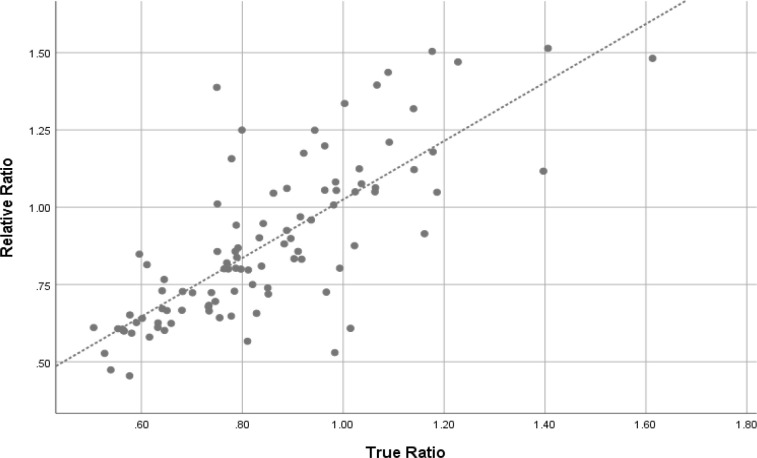
Scatter Plot of Beam-Defined and Diameter-defined Ratios. (r = 0.768, p-value = 0.01)

Pearson’s correlation demonstrated strong, positive correlation between beam-defined and diameter-defined ratios (r = 0.768, p-value = 0.01) (Figure 3). The mean (SD) of beam-defined and diameter-defined ratios for nodule shape of all data were 0.85 (±0.21) and 0.88 (±0.26), respectively (p-value = 0.051). When excluded the equivocal ratios (ratios range from 0.9-1.1), the mean (SD) were 0.78 (±0.23) and 0.81 (±0.27), respectively (p-value = 0.118). 

## Discussion

Our study showed that size and age are independent predictors of malignancy in follicular and Hurthle cell tumors. FC and HC are significantly larger than adenomas with the optimal cutoff at size ≥ 4 cm (OR = 4.5, p-value = 0.006). Several studies mentioned that FC and HCC tend to be larger in size as compared to other types of malignancy but they did not compare them to their benign counterparts (Méndez et al., 2008; Gulcelik et al., 2008; Choi et al., 2009; Ibrahim et al., 2015; Jin et al., 2018). Increasing tumor volume was also reported to increase the risk of malignancy in FN (Sillery et al., 2010). Previous studies on FN cytology reported that size was not a predictor of malignancy, however these studies compared final pathology of malignancy with final benign pathology without subclassification of a specific tumor subtype; e.i. Follicular and papillary carcinomas were considered all together as malignancy (Méndez et al., 2008; Gulcelik et al., 2008; Choi et al., 2009; Parikh et al., 2013; Ibrahim et al., 2015). When it comes to the size, measurement method is crucial. Our study is one of the few studies describing the method of measurement, which we did according to a standard system (Tessler et al., 2017).

Even though increasing age unsurprisingly increases risk of malignancy, we showed the odds ratio of each age group, which could be of additional value for triaging patients based on this basic parameter. We found an optimal cutoff age at 55 year-old (OR = 2.4-3.6, p-value = 0.03).

To the best of our knowledge, our study is the first showing that taller-than-wide shape is not an independent predictor for malignancy in FN and HCN. Taller-than-wide shape has been well accepted to be an important malignant US feature in thyroid cancer. However, most studies on thyroid cancer are weighted toward findings of papillary carcinoma which has different nature both clinically and histologically (Raparia et al., 2009; Yoon et al., 2010; Castro et al., 2011; Ren et al., 2015; Espinosa De Ycaza et al., 2016; Tessler et al., 2017; Yang and Fried, 2018). A previous study using CT comparing shape of FN and nodular hyperplasia found that taller-than-wide shape is significantly more common in FN, irrespective of adenoma or carcinoma (Lee et al., 2016). Several studies have reported that follicular variant of PTC has significantly less taller-than-wide appearance as compared to conventional PTC (Kim et al., 2009; Anuradha et al., 2016; Jeon et al., 2016). Interobserver agreement of taller than wide shape ranges from fair to almost perfect (kappa ranges from 0.282-1) in previous studies.(Chandramohan et al., 2016; Tessler et al., 2017; Grani et al., 2018; Phuttharak et al., 2019; Pang et al., 2019). We believe this variation occurs because of variability in the position of the US probe in real time scanning. We found a near-significant difference between the beam-defined and diameter-defined ratios on the entire data (p-value = 0.051) but after excluding the equivocal ratios (ratios range from 0.9-1.1), the difference was not significant (p-value = 0.118). Angulations of the probe would not have a serious effect on nodules that are strikingly taller than wide. However, for nodules that are equivocal, the different angulation will affect the ratio; hence affect the overall shape. Inspection alone without measurement will cause discordant comments on the same nodule. 

Limitations of this study is that the data is from a single institution and it was collected retrospectively. A large number of patients were excluded due to the incompleteness of data (e.g. both longitudinal and perpendicular views). Nonetheless, our study has one of the highest numbers of follicular and Hurthle cell neoplastic pathology with almost balanced number of adenomas and carcinomas. 

In conclusion, FC and HCC are significantly larger than FA and HCA with a significant cutoff value at 4 cm. Increasing age groups show increasing odds ratios of malignancy. Our study suggests that taller-than-wide shape is not a predictor for carcinoma in this group of patients, in contrast to PTC. We believe that this knowledge will be helpful in more accurately assessing the patient’s risk of malignancy thus better guiding subsequent management.

**Table 1:** Descriptive Data and Univariable Logistic Regression Analysis

		Benign (n=58)	Malignant (n=57)	P-value	Exp (B)	95% C.I.for EXP(B)	
		Mean (SD) or Count (%)	Mean (SD) or Count (%)			Lower	Upper
Gender (Female)		41 (70.7%)	29 (50.9%)	0.031^a^	0.429	0.199	0.925
Age		55.2 (15)	62.6 (16)	0.014^a^	1.032	1.006	1.058
TSH Value (mIU/L)		2.28 (1.45)	2.48 (1.53)	0.585	1.094	0.792	1.513
TSH level	Low	0/37 (0%)	1/32 (3.1%)	0.794	0	0	
	Normal	30/37 (81.1%)	23/32 (71.9%)				
	High	7/37 (18.9%)	8/32 (25%)				
Width (mm.)		22.7 (10.8)	32.9 (18.9)	0.001^a^	1.048	1.018	1.079
Depth (mm.)		19.2 (10.2)	28.9 (17.7)	0.001^a^	1.059	1.023	1.096
Height (mm.)		27.9 (12.2)	40.5 (20.2)	< 0.001^a^	1.047	1.021	1.074
Taller-than-wide		10 (17.2%)	17 (29.8%)	0.115^a^	2.04	0.841	4.951

^a^, Statistically significant for univariable analysis.

**Table 2:** Multivariable Logistic Regression Analysis

	B	S.E.	Wald	df	Sig.	Exp(B)	95% C.I.for EXP(B)	
							Lower	Upper
Age*	0.027	0.013	4.148	1	0.042^a^	1.028	1.001	1.055
Gender**	-0.297	0.471	0.398	1	0.528	0.743	0.295	1.869
Taller-than-wide**	0.324	0.642	0.256	1	0.613	1.383	0.393	4.865
Height*	0.045	0.013	11.433	1	0.001^a^	1.046	1.019	1.073
Width**	-0.001	0.041	0	1	0.989	0.999	0.923	1.083
Depth**	0	0.039	0	1	0.998	1	0.925	1.08

*, Variables analyzed by forward logistic regression (LR) and backward LR methods, **, Variables analyzed by enter method, a Statistically significant for multivariable analysis.

**Table 3:** Odds Ratio of Tumor Size According to Height

Tumor size	OR		95% C.I.	
		P-value	Lower	Upper
1-1.9 cm.	1	0.042		
2-2.9 cm.	2.013	0.195	0.698	5.806
3-3.9 cm.	1.687	0.446	0.439	6.489
≥ 4 cm.	4.5	0.006^a^	1.549	13.07

OR, Odds ratio; ^a^, Statistical significant difference at size equal to or more than 4 cm.

**Table 4:** Odds Ratio of Age

Age (year)	OR	p-value	95% C.I.	
			Lower	Upper
≤ 55	1	0.032		
56-75	2.431	0.033^a^	1.072	5.512
≥ 76	3.647	0.027^a^	1.161	11.457

OR, Odds ratio; ^a^, Statistical significant difference at age more than 55 year-old.

## Author Contribution Statement

A.B. and Z.A. conceived of the presented idea. A.B. developed the theory and performed the computations. A.Z and K.P. contributed to sample preparation. C.D. and D.E. verified the analytical methods. M.R.C, M.S., D.E and B.E. supervised the findings of this work. All authors discussed the results and contributed to the final manuscript.
